# Physical Inactivity and Depression: The Gloomy Dual with Rising Costs in a Large-Scale Emergency

**DOI:** 10.3390/ijerph20021603

**Published:** 2023-01-16

**Authors:** Roberto Codella, Andrea Chirico

**Affiliations:** 1 Department of Biomedical Sciences for Health, Università degli Studi di Milano, 20133 Milan, Italy; 2 Department of Endocrinology, Nutrition and Metabolic Diseases, IRCCS MultiMedica, 20138 Milan, Italy; 3 Department of Psychology of Development and Socialization Processes, “Sapienza”University of Rome, 00185 Rome, Italy

At the end of October of 2022, the World Health Organization (WHO) released “the Global status report on physical activity 2022” [1]—the yearly document on the extent to which governments are implementing recommendations to increase physical activity across all ages and abilities. As usual, the report contains numbers and facts to track the progress on national policies. It is aimed to facilitate the increase in physical activity in terms of infrastructures and promotional actions as a cultural shift is needed. However, what is most astonishing is that this document turned out to be the first-ever report on *physical inactivity*. Defining a concept by its opposite might be quite typical in a heuristic approach; nevertheless, this appeared to be an indicator that lack of physical activity is so globally overwhelming as to become a priority.

## 1. Introduction

Exercise is one of the most transformative things that humans can do for their own physical and mental well-being. Regular physical activity is associated with a multitude of health benefits, from the prevention and the management of non-communicable diseases (NCDs) to reducing the risk of developing several cancers and reducing the symptoms of depression and anxiety [2]. These all-encompassing treasured effects not only reflect individual health status but also rebound at societal, economic, and environmental levels. In fact, enabling people to be more active ignites a virtuous cycle of actions embracing supporting policies, regulatory and technical solutions, mobilizing resources, and securing multisectoral engagement [3].

On the contrary, when these factors are not aligned towards a unifying policy or even one of these factors results inefficient or inadequate, then the entire implementation strategy weakens. As a result, the burden of physical inactivity for society as a whole becomes unsustainable.

## 2. Physical Inactivity Is the Greater Evil

Physical inactivity is a pandemic. It was so a long time before another pandemic occurred due to the spread of SARS-CoV-2. Almost 30% (1.4 billions) of the global adult population does not meet the recommended levels of physical activity according to WHO [4]. Contrary to what one might think, adolescents are indolent too. Eighty-one percent of boys and girls aged 11–17 years do moderate- to vigorous-intensity physical activity for less than 60 min a day. Additionally, physical inactivity is more prevalent in girls than boys in most countries (85% and 77.6%, respectively).

Worryingly, physical inactivity is not only associated with an emerging rise of NCDs but also with a multifaceted encumbrance of indirect health-care costs, productivity losses, and disability-adjusted life-years. Globally, almost 500,000 new cases of preventable chronic diseases will occur between 2020 and 2030 with an estimated expense of nearly US$ 300 billion, around US$ 27 billion annually. With being said that, physical inactivity bears premature deaths [3]. A full understanding of all these deficit estimates is essential for informed decision-making policies [3]. In 2018, WHO launched the Global Action Plan on Physical Activity 2018–2030 (GAPPA) [5] with the intent to raise awareness of the need for accelerated whole-of-government efforts to promote physical activity. By recognizing this as a public health priority, GAPPA has the ultimate mission to reduce by 15% the prevalence of physical inactivity by 2030.

### Under-Powered National Policies

The 2022 report of WHO highlighted that, although 47% of the countries have a standalone national physical activity policy, only 38% of the countries have a policy being actually operational [1]. It is possible that extant policies might be disrupted by the COVID-19 pandemic, augmenting inequities and disparities. However, some countries do not even set a national agenda for physical activity. GAPPA addresses these discrepancies and barriers to help governments maximize the implementation efforts in increasing population levels of physical activity. In doing so, GAPPA identifies four areas of action: systems, societies, people, environment. Intervening synergically (and monitoring the output of these interventions) on these areas may accelerate the policy progress.

Investing in strategies promoting physical activities has a return. For example, according to a recent study [6], if everyone met WHO’s recommendations on physical activity [7], this would increase global gross domestic product by between 0.15–0.24% per year by 2050, worth up to US$ 314–446 billion per year.

Ultimately, the aforementioned report contains five evidence-based recommendations to strengthen policy implementation models: (1) tightening the political leadership with the whole-of-government ownership; (2) being sure to have physical activity in all relevant policies; (3) building capacity in people; (4) enabling data surveillance; and (5) aligning fundings.

## 3. The Major Costs of Physical Inactivity Originate from Depression

As expected, a variety of health indicators are significantly affected by worldwide prevalence of physical inactivity, in terms of direct and indirect costs. Surprisingly, much of this economic burden does not only fall within cardiovascular disease causes. In fact, in Figure 6 of the report, we found that most of the direct health costs attributed to physical inactivity (28%) derive from depression. In detail, Figure 6 shows the distribution in percentage of total number of cases of NCDs and relative costs. Whereas 47% of the new NCDs cases comes from hypertension, depression accounts for 43% of the new NCDs cases. However, hypertension costs come second (22%) with respect to those incurred for the treatment of depression. It is possible that this gloomy picture could have been worsened by COVID-19 itself [8,9]: confinement measures and mobility restrictions might have exacerbated inequities and obstacles to opportunities for being physical active. On the other side, the pandemic of COVID-19 remarked how an active lifestyle is fundamental for mental and physical well-being [10,11].

### 3.1. Small Doses of Physical Activity Can Lower Risks of Depression

Depression is a leading cause of disability burden in developing countries [12] and a common mental health disorder worldwide. While pharmacotherapy and psychotherapy represent currently the elective therapy, their impact is still limited on prevalence, and one third of people with depression remain unresponsive to treatment [13]. Additionally, pharmacotherapy may have adverse side-effects [14] and both pharmacotherapy and psychotherapy cannot resolve physical comorbidities associated with depression [15]. Nevertheless, several modifiable factors can favorably act on depression and they are far from being ascertained. One of these may be physical activity. Moderate evidence sustains a beneficial effect of exercise on depression symptoms [16].

A recent systematic review and meta-analysis, including 15 prospective studies with more than 2 million persons-years, found that practicing regular physical activity, thrice a week, reduces by 25% all the depressive episodes [17]. In detail, physical activity and incident depression were inversely associated in a curvilinear dose–response relationship, with greater differences in risk at lower exposure levels [17]. Compared to inactive people, those fulfilling WHO’s recommendations on physical activity had lower risks of depression. Even below the recommended levels, small doses of physical activity can gain mental benefits and are associated with lower risks of depression. Furthermore, depression incidence is lower in people exercising regularly [18]. Overall, physical activity can be positively considered for prevention of depression, albeit the causative mechanisms are unestablished.

### 3.2. Possible Mechanisms and Types of Physical Activity for Antidepressant Effects

Physical activity elicits an ample spectrum of changes at systemic, cellular and molecular level. Beyond its notorious anti-inflammatory effects [10], exercise has been controversially indicated as a natural anti-depressant [19]. Fit and trained people show resistance against depression and other psychiatric disorders, like anxiety and stress [20]. Several neurotransmitters and neurotrophic factors, within the central reward circuitry [21], are critical for both the pathophysiology and treatment of stress-related illnesses. Doing exercise is a naturally reinforcing and rewarding activity [22].

To summarize, the putative exercise-triggered mechanisms would encompass:

(a) A virtuous cycle in a healthy lifestyle combining physical exercise and sociality [2]. That means that physical activity exerts a synergistic, dragging-effect on healthy sociality [23]: people who exercise regularly can await positive feedback from their environment and social interaction. Through psychosocial leverages, physical activity may positively affect sub-domains such as self-esteem, self-efficacy, and social support [24].
(b) The involvement of the neuroendocrine system including the effects on inflammation, oxidative stress, and the amount of beta-endorphins and monoamine concentrations [25];
(c) The development of the hippocampus volume with the growth of nerve cells stimulated by brain-derived neurotrophic factors (BDNF) [26]. The hippocampus is an area primarily implicated in processes relevant to depression [27], such as emotional processing [28], and stress regulation. Exercise modifies brain connectivity leading to augmented neuroplasticity. These elicited changes regard neurotransmission, neurogenesis and synaptogenesis. In fact, it seems that depression disrupts consistently several key brain regions by: (i) affecting cerebral blood flow (Cooper et al., 2019); and (ii) diminishing the number of BDNF.

Furthermore, the virtuous association between depression and physical activity (see paragraph 3.1) appears to vary among activity types. In a Brazilian National Health Survey [29] involving 88,522 adults aged 17–107 years, cycling, team sports, and outdoor walking/running were associated with fewer depressive symptoms. These individuals had no previous history of depression.

Meditation exercises were associated with lower odds of depression among those with no precedent diagnosis of depression. Adults performing mindful exercises showed lower mental health stress than inactive subjects [30]. In numerous studies, yoga routines procured a relieved psychological burden and a greater well-being with respect to control individuals [31], even if in older adults [32].

Outdoor activities like cycling and walking/running bear psychological benefits such as lower prevalence of depression, decreased levels of stress, and improved mental health [33]. A higher exposure to sunlight and green areas is associated with rewarding and pleasant experiences [34]. Additionally, the concept of outdoor educational system is relevant for children’s cognitive and physical development (other than protecting them from overweight and obesity). In another study, cycling (15–25 min/week) below the ventilatory threshold exerted a therapeutic effect for depression: it improved social, cognitive functioning, and quality of life in patients diagnosed with major depressive disorder [35]. Other meta-analyses indicated that low cardiorespiratory fitness (CRF), an indicator of physical inactivity, bore a 64% higher risk of depression than high CRF across around 4 million individuals per year [24,36].

Altogether, these findings suggest that governments policies should promptly consider implementing supportive environments and models capable to positively impact physical activity engagement.

## 4. Conclusions

Health systems and communities must immediately convey their willingness and resources for the promotion and the increase of the physical activity levels at large scale. Physical activity will bring social, environmental, and economic benefits.
